# Experiences, perspectives and values of Indigenous peoples regarding kidney transplantation: systematic review and thematic synthesis of qualitative studies

**DOI:** 10.1186/s12939-019-1115-y

**Published:** 2019-12-30

**Authors:** Rachael C. Walker, Sally Abel, Annie Reynolds, Suetonia C. Palmer, Curtis Walker, David C. Tipene-Leach

**Affiliations:** 1 School of Nursing, Eastern Institute of Technology, 501 Gloucester Street, Taradale, Napier, Hawke’s Bay 4112 New Zealand; 2 Kaupapa Consulting Ltd, Napier, 4110 New Zealand; 3 Department of Medicine, Hawke’s Bay District Health Board, Hastings, 4130 New Zealand; 4 Department of Medicine, University of Otago, Christchurch, 8140 New Zealand; 5 Department of Medicine, Midcentral District Health Board, Palmerston North, 4442 New Zealand; 6 Research and Innovation Centre, Eastern Institute of Technology, Napier, 4112 New Zealand

**Keywords:** Indigenous, Kidney transplant, Qualitative, Systematic review

## Abstract

**Background:**

Kidney transplantation is considered best practice treatment for end stage kidney disease (ESKD), however Indigenous patients are substantially less likely to receive either a deceased or live donor kidney transplant than non-Indigenous patients. We describe Indigenous peoples’ experiences and perspectives including traditional values around kidney transplantation to inform international transplant programs.

**Methods:**

We conducted a systematic review of qualitative studies involving Indigenous adults who have experience with or perceptions of kidney transplantation. We searched MEDLINE, Embase, PsychINFO, and CINAHL, in conjunction with analysis of Google Scholar and reference lists of related studies till July 2019. We utilised thematic synthesis to analyse data. Completeness of reporting in studies was evaluated using the Consolidated Criteria for Reporting Qualitative Studies (COREQ) framework.

**Results:**

Eight studies involving 225 Indigenous participants were included. Five themes were identified: *strong desire for transplantation* (seeking normality and freedom from dialysis, wanting to reduce burden of disease within community); *lack of partnership in shared decision-making* (receiving inadequate information, ineffective communication); *barriers to live kidney donation* (difficulty asking, apprehension about impact on donor, avoiding additional financial burden and fear of complications); *cultural considerations* (influence of traditional values and beliefs, reconciling traditional values with pragmatic need); and *experiencing lack of cultural competence in clinical care* (struggling with prejudice and ignorance, mistrust of clinicians and health system).

**Conclusion:**

Indigenous participants had a strong desire for a kidney transplant and recognised the need for more readily available kidney transplants for others in their communities with ESKD. However, they faced prejudice and a lack of cultural competence by health workers as well as wider barriers to transplantation in systems that did not support effective and culturally appropriate delivery of information and care. Traditional cultural values also influenced decisions regarding kidney transplantation but such values were moderated when considering transplantation. Transplantation programs need to identify and mitigate barriers, such as the financial burden, promote cultural safety and incorporate traditional values into the promotion of transplantation in order to address inequitable transplantation rates.

**Registration:**

Not applicable.

## Background

Kidney transplantation is considered optimal treatment for End Stage Kidney Disease (ESKD). Transplantation is associated with markedly improved clinical and patient-reported outcomes compared to dialysis, including increased life expectancy and quality of life, and decreased patient and health system costs [1–3]. Inequitable access to kidney transplantation for Indigenous populations has been a sustained phenomenon over decades [4] and the causes of this differential access to transplantation are incompletely understood. Non-Indigenous populations have substantially lower rates of ESKD and experience greater access to the waiting list for kidney transplantation even when accounting for socioeconomic factors, geographical location and comorbidity [4, 5], suggesting that systemic barriers play a role in the inequitable rate of kidney transplants.

A number of barriers to kidney transplantation in Indigenous populations have been previously identified, including a lack of suitable donors, socio-economic factors, remoteness and low levels of health literacy [6, 7]. With particular regard to Indigenous patients with ESKD, identified barriers also include distrust of health care systems, lack of knowledge of kidney transplantation processes and discrimination [5]. Despite this previous research exploring these barriers, there have been few studies exploring whether the perspectives and cultural values of Indigenous individuals can enhance understanding of these inequities and inform transplantation care and improvement.

Thematic synthesis of qualitative data from multiple studies can provide detailed and diverse evidence about peoples’ experiences, perspectives, values, attitudes, knowledge and beliefs across different healthcare contexts, countries and cultures. In this systematic review of qualitative studies we aimed to answer the research question, “What are the experiences, perspectives and values of Indigenous peoples regarding kidney transplantation**”**, in order to identify systemic barriers and understanding of cultural values that may influence the uptake of kidney transplantation by Indigenous peoples.

## Methods

We followed the Enhancing Transparency in Reporting the Synthesis in Qualitative Research (ENTREQ) framework [8] (Additional file 1).

### Data searches

Pre-planned systematic electronic searches of databases, including MEDLINE, EMBASE, PsycINFO, and the Cumulative Index to Nursing and Allied Health Literature (CINAHL), were conducted from database inception to June 17, 2019 without language restriction. The search strategy is provided in online Additional file 2. We also searched Google Scholar and reference lists of relevant articles. Three authors (RCW, AR and SA) independently screened the citations and excluded those that did not meet the inclusion criteria. The full texts of potentially relevant studies were then assessed for eligibility by the same three authors. Any discrepancies were discussed with author SCP.

### Study inclusion and exclusion criteria

**Fig. 1:**
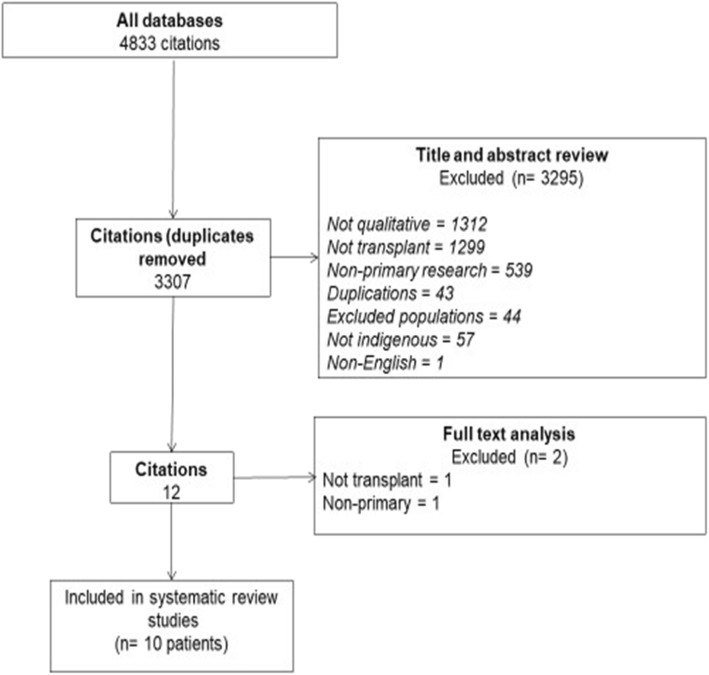
Prisma diagram of study searches and screening

Qualitative studies in which Indigenous adults aged 18 years or older who expressed views or experiences about kidney transplantation were included. Studies were ineligible if they did not include studies of Indigeous adults or were not specific to kidney transplantation. We excluded quantitative and epidemiological studies, non-primary research, clinical guidelines, economic studies and non-English articles (Fig. 1).

### Study quality assessment

The comprehensiveness of reporting of each primary study was assessed according to the adapted Consolidated Criteria for Reporting Qualitative Research (COREQ) framework. This framework includes domains specific to the research team, study methods, study setting, analysis, and interpretations [9]. Three authors (RCW, AR, & SA) independently assessed each study and any discrepancies in assessment were resolved through discussion with a fourth author (SCP).

### Data synthesis and presentation

We used thematic synthesis as described by Thomas and Harden [10]. One author imported text and participant quotations under the Results/Findings or Discussion section of each study into HyperRESEARCH (version 3.0.3; ResearchWare Inc., 2009) software. Text and quotes were extracted from primary studies if they aligned with the research question. Three authors (RCW, AR and SA) independently performed line-by-line coding of the primary studies, conceptualized the data, and inductively identified concepts. After this, all authors discussed the concepts and developed themes and subthemes. Conceptual links among themes were identified by all authors using a mind-mapping approach to extend the findings offered by the primary studies and develop an analytical thematic schema. Three authors independently reviewed the primary studies, preliminary themes and analytical framework. All authors then convened to discuss the emerging themes and ensure that coding captured all relevant issues and reflected the primary data. Subsequent revision of themes were discussed iteratively among all authors.

## Results

### Literature search

The electronic search yielded 4833 citations, of which eight studies (reported in 10 publications) were eligible. These included 225 Indigenous participants (Fig. 1) in Australia, New Zealand, Canada and the United States. Participants included 171 potential and 11 actual recipients and 42 potential (general population) and one actual donors. Characteristics of included studies are listed in Table 1.

**Table 1:** Characteristics of Included Studies

Study	Country	No. Indigenous participants (n)	Participant group(s)	Age range (years)	Methodological framework	Data collection	Analysis e.g. content, framework, thematic, GT	Topic
Anderson et al. 2008* [11]	AU	146 Aboriginal & Torres Strait Islanders	ESKD pts	(18–65)	Interview study -life story narrative	In depth narrative style face to face interviews	Thematic content analysis	Understanding of their chronic kidney disease and attitudes to treatment
Bennett et al. 1995 [12]	AU	11 Aboriginal & Torres Strait Islanders	Transplant recipients	NS	Exploratory qualitative	In-depth interviews	NS	Social and cultural factors in dialysis and transplantation
Davison & Jhangri 2014 [13]	CA	21 First Nations	General population	18+ (Age criteria)	Multiple case study	Semi-structured face to face interviews	Critical realism	Attitudes toward organ transplant and donation
Devitt et al.* 2017 [14]	AU	146 Aboriginal & Torres Strait Islanders	ESKD pts	18–65	Interview study -life story narrative	Narrative style face to face interviews.	Thematic	Views of transplant and organ donation
Fahrenwald & Stabnow 2005 [15]	US	21 Oglala Lakota Sioux	General population	21–82	Ethnographic Social-ecological	In-depth Interviews	Thematic	Sociocultural patterns influencing decisions about organ and tissue donation
Jones & Cornwall** 2018 [16]	NZ	9 Māori	ESRD pts	Mean 49.8 (Total Qualitative Sample)	Qualitative descriptive	Face to face semi-structured interviews	Thematic	Factor influencing decision-making among ESRD pts. considering approaching family or friends for a kidney
Jones** 2017 [17]	NZ	9 Māori	ESRD pts	23–68 Mean 49.8 (Total Qualitative Sample)	Qualitative descriptive	Face to face semi-structured interviews	Inductive thematic	Factor influencing decision-making among ESRD pts. considering approaching family or friends for a kidney
Keddis 2019 [18]	US	12 Native American	ESKD presenting for kidney transplant evaluation	Mean 50.1 (+/−8.65)	Exploratory qualitative	Semi structured interviews	Grounded theory & inductive thematic analysis	Perceptions & attitudes about kidney transplant
Martin 2013 [19]	NZ	4 Māori	ESRF waitlisted for kidney transplant	19–77 (Total Qualitative Sample)	Pragmatic mixed methods -including qualitative interviews	Semi structured face to face interviews	A priori and inductive thematic analysis	Barriers to live donor kidney transplant
Smith 2015 [20]	CA	1 First Nations	Donor	NS	Autoethnography	Journal records	NS Narrative	Lived experience of kidney donation

### Comprehensiveness of reporting

The comprehensiveness of study reporting was variable (Table 2). No study reported on whether relationship of interviewer was established prior to interviews, whereas all studies presented participant quotations. Participant selection strategy was described in nine publications. Theoretical saturation, defined as when few or no new concepts are identified in subsequent data collection, was reported in five studies. Member checking (obtaining feedback from participants on the preliminary findings) was reported in only one study, whereas researcher triangulation in data analysis was reported in 6 (60%) studies. One thesis included in this review [19] did not include participant quotations identifiable by ethnicity, however the author was contacted and reported that all themes in the study represented perspectives of Indigenous participants.

**Table 2:** COREQ Assessment

Item	Studies reporting each item	Number of studies (%)
Personal Characteristics		
Interviewer / facilitator identified	[11–20]	10 (100%)
Occupation of the interview of facilitator	[11, 17, 19, 20]	4 (40%)
Experience or training in qualitative research	[11, 13]	2 (20%)
Relationship with participants		
Relationship established prior to study commencement		0 (0%)
Participant Selection		
Selection strategy *(*e.g. *snowball, purposive, convenience, comprehensive)*	[11–19]	9 (90%)
Method of approach or recruitment	[11, 14, 15, 17–19]	6 (60%)
Sample size	[11–20]	10 (100%)
Number and/or reasons for non-participation	[13, 17–19]	4 (40%)
Setting		
Venue of data collection	[12, 13, 15, 18]	4 (40%)
Presence of non-participants *(*e.g. *clinical staff)*	[13, 18]	2 (20%)
Description of the sample	[13–20]	8 (80%
Data Collection		
Questions, prompts or topic guide	[14–18]	5 (50%)
Repeat interviews / observations		
Audio / visual recording	[11, 13, 14, 16–19]	7 (70%)
Field notes	[13, 17, 19]	3 (30%)
Duration of data collection (interview of focus group)	[12–19]	8 (80%)
Data (or theoretical) saturation	[13, 16–19]	5 (50%)
Data Analysis		
Researcher/expert triangulation (multiple researchers involved in coding and analysis)	[11, 14–18]	6 (60%)
Derivation of themes or findings *(*e.g. *inductive, constant comparison)*	[11, 13–19]	8 (80%)
Use of software *(*e.g. *NVivo, HyperRESEARCH, Atlas.ti)*	[11, 13, 14, 16, 17, 19]	6 (60%)
Member checking (participant feedback on findings)	[18]	1 (10%)
Reporting		
Participant quotations or raw data provided *(picture, diary entries)*	[11–20]	10 (100%)
Range and depth of insight into participant perspectives *(thick description provided)*	[11–20]	10 (100%)

### Synthesis

**Fig. 2:**
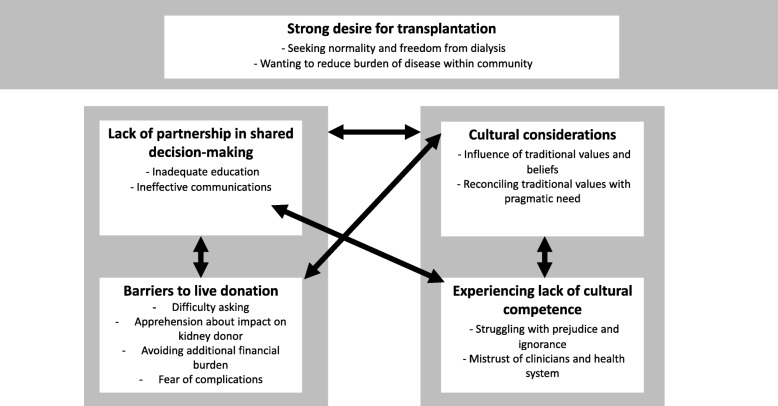
Thematic schema of Indigenous participants perspectives, experiences and values of kidney transplantation. We found that participants recognise the pressing need for more readily available kidney transplants, both for themselves and others in their communities with ESKF. However, they faced prejudice, lack of cultural competence and barriers to transplantation in systems that did not support effective and culturally appropriate delivery of information and care. In particular, participants felt that clinicians often did not understand or acknowledge their cultural beliefs. Although traditional values and beliefs influenced views and concerns regarding transplantation, many acknowledged these alongside the need to be pragmatic about kidney transplants. Finally, participants reported profound difficulty asking others to donate because of the enormity of the request, future potential of obligation to the donor, and the lack of viable donors in their families. They also expressed concern about complications for recipients and the financial burden of both receiving and donating a kidne

We identified five themes: Strong desire for transplantation, lack of partnership in shared decision-making, barriers to live kidney donation, cultural considerations, and experiencing lack of cultural competence. Selected quotations to illustrate each subtheme are provided in Table 3. A thematic schema of the relationships between themes and subthemes is presented in Fig. 2. We found all main themes were represented by donor and recipient participants and, unless otherwise stated, sub-themes pertain to both potential and actual recipients and donors.

**Table 3:** Selected Participant Quotations

1. Strong desire for transplant		
Seeking normality and freedom from dialysis	“I’m tired of being sick. I don’t want to be tied down for 4 h three times a week” [18] “I’d like any kidney, as long as it would keep me alive, I could get back to [home] then ... My family depends on me.” [12] “I can’t go [home] and I’m really missing my friends and my family … I really want to be able to go home” [14] “My brother in law is waiting for a kidney transplant …. He has that dialysis and it makes him weak. He can’t do much of anything anymore” [15]	[12–20]
Wanting to reduce burden of disease within community	“Four years ago I decided that I wanted to be an organ donor. Our elders are dying because of these things and we can do something about that” [15] “Organ and tissue donation is essential and we need it badly on the reservation. People are waiting for kidneys you know” [15] “From the time of first diagnosis, I had volunteered myself as a kidney donor and was very keen about a pre-emptive transplant” [20]. “It would make other people live … you’d be saving somebody’s life … they’d have their life back” [13] “Attitudes need to change, because your own people are needing these body parts” [17]	[13, 15–17, 20]
2. Lack of partnership in shared decision-making		
Receiving inadequate information	“We need information because I think some people don’t always understand how they would be helping other people. You need to talk to the tribal leaders and make videos and booklets that teach about these things. Our health committee should help too. We need to get the word out in meetings and on the local radio” [15] “There probably would be a little bit of fear just because there’s not enough education on it “ [14] “I didn’t really get much information at all. It could have been much better than it was … Now it’s two years later and I’m just starting to find out about transplant” [14] “They should come around and keep you informed, I think, I seem to be starved of information about what is happening inside.” [11]	[11, 14, 15, 18, 19]
Ineffective communication	“They [staff] don’t give it [information] the right way … When they come across like that everyone’s too scared to ask them questions why, so then they just shut up and think, “Well I’ve been told this, so that must be it” [14] “[The doctors] started telling me what’s going on and that. But they had a very funny way of communicating with people. When they talk to us they need to bring it down and explain it to us.” [11] “I don’t know how to talk to the nurse or doctor. He comes down here and just checks out how we’re looking after our body. It’s not enough time” [11] “There’s a whole lot of us who just don’t understand what’s ging on. They know though, the doctors and the nurses know, but they don’t tell us. They don’t talk with us and we’re oblivious” [11]	[11, 14–17]
3. Barriers to live kidney donation		
Difficulty asking	“It’d be like asking for money, in a way. It’s not an easy thing for a donor to do – there is a risk to their health, there’s the surgery and recovery that they have to go through, so it’s not...it’s not a minor request, it’s...it’s pretty major” [17] “No-one’s offered...like I’m from a family of nine, and none of them have offered me a kidney. So if they’re not gonna offer, I’m sure as heck not gonna ask” [17] “I wouldn’t mind if it [LKD offer] was genuine - from the heart you know … that’d be fine. But I feel with the younger brother, maybe he’d be on my back.” [14]	[14–17, 19]
Apprehension about impact on donor	“I also think that many families … do not want the other person to be harmed” [17] “If my Pa donates, and then he dies on the day, well then it’s all on me” [17] “I mean, who knows...In years down the [track], their kidneys might fail and [but for] that one kidney that you might take off them, they could be alive” [14]	[12, 14, 16–19]
Avoiding additional financial burden	“I was still employed on the island, but I had to miss a lot of work. Moreover, the expenses involved in transportation, accommodations, and meals were extremely distressing” [20] It was very tempting at times to feel desperate as if all odds were against going through the transplant, given the costs and geographical travelling challenges [20].	[18–20]
Fear of complications	All I know is that when I was staying at the hostel here a lady came down from Alice Springs for a transplant and when she went back a couple of months ago, she passed away. Yeah that’s what I’m scared of” [14] “What if things went wrong, what if one of us dies during the surgery, what if the kidney fails …? My mind was in a spin” [20] “Like a double edged sword, there is health now, but also the fear of illness recurring and of transplant failure and rejection.” [17]	[12, 14, 16, 17, 19, 20]
4. Cultural considerations		
Influence of traditional values and beliefs	“If there was any trouble, it would be a throw-back from that transplant … just as if a spirit had taken over an action” [12] “I was made with the parts I have and that’s the way I want to go and I wouldn’t donate my parts either because they’re mine not anybody else’s. You come into this world with your own parts and you leave with your own parts” [13] “Pacific Islanders and Māoris are probably the worst for actually giving up body parts...and yet we’ve probably got the biggest demand out there for it...a lot of that is our own cultural beliefs, you know, that you go into the earth all in one piece, and all that, but, yeah … probably need to do some more work around our people’s beliefs.” [17] “It is kinda taboo to take another person’s body part and put them in yours … A lot of elders don’t approve of it … why do you want to bring any kind of weirdness into your family, evil kind of thing.” [18] “Because God is in each of us then we are related and that’s why we call it mother earth and are all related in that way. We need to preserve that spirit and (organ donation) gives a true understanding of what kindness is all about, the miracle of healing.” [13]	[12, 13, 15–18]
Reconciling traditional values with pragmatic need	“No, some may have [reservations], but there is that many people with renal disease that that should overcome any cultural things.” [14] “If I’m gonna get well, I gotta get (a kidney) … I’ll do a karakia (prayer), and make use of the new one” [17] “Every Native American culture kinda frowns upon the fact that you are getting a kidney from a cadaver … For me it is a lifesaving procedure. It is giving you your life back and I’m very good if a get a cadaver.” [18]	[12–17, 20] [18]
5. Experiencing lack of cultural competence		
Struggling with prejudice and ignorance	“Awareness at the hospital is important. They don’t know much about our beliefs.” [15] “I think it’s a cultural thing too. They [renal staff] don’t know whether it’s culturally appropriate to even ask that sort of thing” [14] [Someone] should tell the doctor because some Aboriginal people who are dialysing, they are finding it very hard from our own family, because that’s what the ancestor say. They (family members) say, if they give us a kidney and we die middle of that, maybe after 9 years, or whatever, we pass away and we’ve got their kidney. They think we will haunt them … And no-one has explained that to the doctors” [14]	[14, 15]
Mistrust of clinicians and health system	“I’m very sceptical right now. I believe in tradition. I’m on apole where I could fall either way. It just seems like people’s bodies are kept for longer periods of time [at the hospital] if they are organ donors and I don’t think I want my body to be held up. I want it to be put to rest.” [15] “If you die up here they don’t look at those things (traditional beliefs and requests regarding kidneys). They don’t look at your driver’s liscence. They just send you to the mortuary” [15] “I blamed the doctor because they the one made me think nothing, diabetic and kidney … ..I still blame them [for not telling me earlier]” [11] “You don’t go knocking on their (health professionals) door, [it’s a] danger one … The door is locked. They sit behind closed doors” [14] “I have no idea either [if I am on a transplant list] – I don’t even know that. Well to tell you the truth, I don’t even know what transplant they’re talking about – they just say ‘transplant’, you know ‘kidney’” [14]	[11, 14, 15]

#### Strong desire for transplantation

##### Seeking normality and freedom from dialysis

There was a strong desire for access to kidney transplantation amongst Indigenous participants. Those who had received or were about to receive a kidney identified profound benefits for themselves, family and community [12–14, 16–20]. Transplantation was considered the only opportunity to be released from the restrictions of dialysis and to reclaim independence, freedom and improvements in their quality of life. Having a functioning kidney enabled a return to ‘normality’ and a resumption of family and cultural responsibilities [12–15, 18–20]. Patients required to live considerable distances away from their homelands and supportive communities to receive regular dialysis were especially keen to be transplanted to enable a return to home [11, 12, 18, 20].

##### Wanting to reduce burden of disease within community

The principal motivation for both actual and potential kidney donors was the desire to give. Such giving meant they would be “saving somebody’s life … they’d have their life back” [13]. Some saw saving a life as ensuring cultural continuity [20]. Participants were aware of the increasingly high need for kidney transplants within their communities and that a donation could provide significant benefits to those in need [16, 17]. Those who had first-hand knowledge of a loved one’s need for a kidney were particularly keen on donation [13–15, 20].

#### Lack of partnership in shared decision-making

##### Receiving inadequate information

Indigenous participants reported insufficient efforts made to provide information. They expressed lack of sufficient information about kidney transplantation and its processes at both community and individual levels to enable confident decision-making about transplantation assessment for donors and recipients. They reported very little, if any, awareness of or information about kidney transplantation provided to communities who therefore had insufficient knowledge about the possibility and processes to donate. In addition, Indigenous potential recipients reported not receiving timely information about their eligibility for kidney transplantation as a treatment option, impeding the actions required to commence assessment [11, 12, 14, 15, 18, 19]. One participant reported not being informed about transplant as an option until “about two years after [starting] my dialysis treatment” [14].

##### Ineffective communication

A key barrier to partnership in shared decision-making was ineffective communication by health professionals because of the manner in which information was imparted. This often arose from use of medical jargon that impeded knowledge transfer [11, 14–17]. Potential transplant recipients and donors reported feeling too intimidated to ask for clarification, which prevented them from actively pursuing transplantation as a treatment option [14, 15]. Participants for whom English was not their first language reported language as a barrier and experienced inadequate strategies used in clinical care to ensure that core issues regarding transplantation were communicated effectively [11, 14].

#### Barriers to live donation

##### Difficulty asking

Potential recipients reported particular difficulty in asking family or others to be assessed as a directed kidney donor [14–17, 19]. Seeking a donor was especially challenging when there was a high level of shared medical comorbidity within families and communities, increasing the anticipated risk to the donor. This led to limitations in available people to ask as well as increased concern that a request to a potential donor was too big a request to make of others. Some participants hoped ideally that someone would offer without being asked so would wait for a potential donor to come forward [15–17, 19]. Many participants described reciprocity as an important cultural value for Indigenous peoples and another source of reluctance was not wanting to feel indebted to the donor, particularly if they were not confident about the proposed donor’s motive [14].

##### Apprehension about impact on donor

Concerns about the impact of donating a kidney on the health of the donor were expressed by potential recipients. They worried about the possible negative health consequences for the donor from the surgery or the loss of a kidney and that the donor may need their own donated kidney in the future [12, 14, 18, 19]. Some also worried that younger (more worthy) family members may later require their potential donor’s kidney [16, 17].

##### Avoiding additional financial burden

Concern about the financial burden experienced by families involved in both kidney donation and transplantation was also expressed by participants. Direct costs included, loss of income from time off work to undergo the medical and recovery processes, and expenses associated with travel, accommodation and food if one had to live away from home to be near medical facilities [18–20]. These were particularly onerous if they were already struggling financially and/or from rural close-knit Indigenous communities who needed to be in town for long periods [20].

##### Fear of complications

Participants in general held concerns about the safety of the transplantation surgical procedure, which were exacerbated by reports of poor transplantation outcomes by community members [12, 14, 16, 17, 19]. Specific fears of adverse outcomes such as “psychic fragmentation” because of “serious cultural transgression” [14] were voiced by some participants. Those returning to remote areas feared insufficient medical care after transplantation could lead to poorer clinical outcomes [12, 20]. This included marked concerns about kidney transplant failure, leading to a return to dialysis and substantial time spent away from home and trying to find another donor [14, 16, 17, 20].

#### Cultural considerations

##### Influence of traditional values and beliefs

Traditional values and beliefs were seen as both inhibiting and supporting kidney transplantation. A traditional value commonly described by Indigenous participants was the importance of having an ‘intact body’ at death, a belief unsupportive of organ transplantation in general [13, 15–18]. Adherents to this value tended to be those who did not have direct personal experience of dialysis and community elders, who could influence community views and individual decisions around transplantation [15–17]. A few groups were also wary of transplantation, believing that traits of the donor could influence the recipient in some way [12, 18]. By contrast other cultural values directly supported the act of giving or receiving a kidney as a form of spiritual interconnectedness [12, 13].

##### Reconciling traditional values with pragmatic need

Participants reported feeling tension between traditional values and beliefs that precluded kidney transplantation versus the desire for transplantation to prevent death or very limited quality of life on long-term dialysis. However, while traditional beliefs were considered legitimate and were respected, those who faced the reality of ESKD described a pragmatic need to balance their beliefs with the desire for transplantation [13–18, 20]. Those who were concerned about the implications of cultural transgressions used specific strategies (such as prayer or ritual) to mitigate any potential negative effects [12, 18].

#### Experiencing lack of cultural competency in clinical care

##### Struggling with prejudice and ignorance

Participants who had engaged with the health system reported discomfort and some lack of cultural safety in the care they received. This was expressed as feeling intimidated by the system, believing their cultural beliefs were not well understood or respected by health professionals and that clinicians’ poor communication was based on negative or prejudiced judgments about the person. Also mentioned was a lack of culturally appropriate resources and education about kidney transplantation. Participants reported a need for better cultural understanding by health service providers and improvements in kidney donation cultural protocols [14, 15].

##### Mistrust of clinicians and health system

Closely linked to the above experiences was a general lack of trust in health professionals and the health care system based on previous experiences where participants felt they had been treated unfairly. In addition to mistrust and fear that cultural views would be misunderstood, ignored and overridden, was ambivalence about the biomedical model in general and fears of cultural incompetence around the way the healthcare system dealt with donated live kidneys and deceased donor kidneys [15]. Exacerbating this sense of mistrust was feeling patronised and poorly informed by health professionals about the availability of kidney transplant as a treatment option, a sense that information was being deliberately withheld and a perceived lack of transparency about the allocation of kidneys on the deceased donor list [11, 14, 15]. In one study involving 137 Indigenous patients approximately a quarter did not know or were under a misunderstanding about their transplant status [14].

## Discussion

This systematic review has synthesised the experiences, perspectives and traditional values related to kidney transplantation amongst Indigenous peoples of four Western nations. Our search identified 8 studies (10 papers) involving 225 Indigenous people from Canada, United States, Australia and Aotearoa/New Zealand.

We found that participants had a strong desire for a kidney transplant and recognised the need for more readily available kidney transplants as a fundamental requirement to improve survival and quality of life as well as to sustain community strength and longevity in face of the impact of ESKD. However, Indigenous participants faced prejudice and a lack of cultural competence by health workers as well as wider barriers to transplantation in systems that did not support effective and culturally appropriate delivery of information and care. In particular, participants felt that clinicians often did not understand or acknowledge their cultural beliefs. Traditional values and beliefs influenced views and concerns regarding transplantation and, while many acknowledged these, strongly evident was the need to be pragmatic about kidney transplantation. Finally, participants reported profound difficulty asking others to donate because of the enormity of the request, future potential of obligation to the donor, and the lack of viable donors in their families. They also expressed concern about complications for recipients and the financial burden of either receiving or donating a kidney.

A previous general population systematic review and thematic synthesis exploring patients’ views of kidney transplant wait-listing included one minoritized population study but did not specify any Indigenous ones [21]. It identified a few themes similar to those in our review. In that review, participants also sought transplantation to regain normality and avoid dialysis. Subthemes of eligibility enigma, and uncertainty about what determined wait listing were also identified. Some studies included in that review found participants felt they were deprived of the opportunity to be listed and suspected inequities existed. However, these were predominantly perceived as due to age and co-morbidity [22, 23] with only the one minoritized study of African American people seeing this as resulting fromrace [24]. A further systematic review synthesised perspectives regarding live kidney transplantation [25] from more diverse populations. Although studies were described according to country, not ethnicity, overall 41% of participants were reportedly from ethnic minorities (although there was no explicit mention of Indigenous populations). That review identified more themes similar to ours, including aversion to dialysis; seeking better graft survival; concerns regarding donor health, donor regret and donor financial and other inconvenience; insufficient information delivery, particularly for ethnically and linguistically diverse and minoritized patients; and the need for resources to be more culturally sensitive. Many of the themes in these two reviews that were similar to our own appear to have been derived from studies involving minoritized populations.

Smedley et al. [26] theorise that ethnic health disparities arise from a complex combination of health professional, health system and population factors**.** We found our inductively derived themes fitted well into this framework. The poor delivery of key information reported by participants and the feeling that Indigenous cultural beliefs and values were not understood or respected both point to a lack of cultural competence by health professionalss. But this is also indicative of wider institutional racism within health systems. Institutional policies, processes and practice typically service the majority dominant culture and, as described here, efforts are not specifically geared to ensure Indigenous populations were informed or able to navigate the transplant process adequately.

The unfavourable social determinants of health experienced by most in Indigenous communities are also significant. This prohibits both donors and recipients progressing easily towards transplantation, an issue that has been identified in many countries [6, 27–29]. Those from more socioeconomically disadvantaged groups are less likely to receive a living donor kidney transplantation, a disparity not observed in cadaveric donation [27]. Similarly, racial disparities in access to live donation have also been found to be strongly influenced by financial barriers [29]. Many countries have acknowledged the financial burden for donors and instituted donor reimbursement payments for loss of income, although this may not fully address the issue for those in more deprived groups. In a recent study from America, the authors performed a cost-benefit analysis of government compensation of kidney donors. They included the savings to society of kidney recipients not requiring dialysis and also estimated the monetary value of the longer and healthier lives of kidney recipients and found the benefits to exceed the costs by a factor of three [30].

Previous studies have found that targeted programs that acknowledge traditional values, include families, are supportive of well-informed health decision-making processes, and promote access to live kidney donation [31, 32] will facilitate increased kidney transplantation. Our review suggests that active incorporation of traditional values and beliefs into a proactive pro-donation agenda will help to build more Indigenous patient-centred and culturally appropriate programs. Traditional beliefs are not static; culture is dynamic and changes over time [33] and this is reflected in the pragmatic responses reported by some participants, but not necessarily the views of clinicians treating them [34]. Future programs need to promote working together with elders and other knowledge holders to develop responses to the challenges and needs around transplants that both honour tradition and allow flexibility.

### Strengths and limitations

Our review has a number of strengths. We performed a comprehensive search and independent assessments of study reporting. We synthesised studies from both actual and potential recipient and donor experiences, which generated broader insights into patient perspectives. Some studies, however, had methodological limitations (only half reported data saturation and none reported member checking) which may reduce confidence in the findings. A limitation of this review, like other thematic syntheses based on qualitative data, is that we only were able to analyse the data included in the primary studies’ publications, apart from two studies where we were able to access the full thesis. Another limitation was that there was only one actual donor. There are possibly also limitations in comparing studies from countries with markedly different health care funding systems, with differing socioeconomic conditions and where kidney transplant is newly accessible.

## Conclusions

Indigenous participants both desired a kidney transplant for themselves and recognised a pressing need for more readily available kidney transplants for others in their communities with ESKD. The prejudice and a lack of cultural competence in the workforce worked against such outcomes, as did the institutional barriers to transplantation in systems that did not support effective and culturally appropriate delivery of information and care. Traditional cultural values may impinge upon decisions regarding kidney transplantation, but participants were well able to reconcile traditional belief with their pragmatic need, hence adopting an openness towards and indeed a strong desire for transplantation. Transplant programs need to identify and incorporate these traditional values in order to promote transplantation and address inequitable transplantation rates. Programs also need to explore ways to overcome barriers, such as the financial burden for both donors and recipients, and ensure that clinicians are culturally safe in their care delivery.

## Supplementary information

**Additional file1:**

**Additional file2:**

## Data Availability

Not applicable.
