# Supplementary material for: Experiences, perspectives and values of Indigenous peoples regarding kidney transplantation: systematic review and thematic synthesis of qualitative studies
Source: Int J Equity Health. 2019 Dec 30;18:204. doi: 10.1186/s12939-019-1115-y (PMC6937677; doi:10.1186/s12939-019-1115-y)
Supplement: Supplementary file 2 — Additional file2: [file 12939_2019_1115_MOESM2_ESM.docx]

Additional file 2. Search strategy

Order Search terms - Searches run 19 June 2019

**Scopus**

1. haemodialysis OR hemodialysis
2. dialysis OR predialysis
3. kidney OR renal
4. neph*
5. 1 or 2 or 3 or 4
6. indigen*
7. minority
8. ethnic
9. maori
10. "pacific islander”
11. aborigin*
12. torres
13. hawaii*
14. inuit
15. eskimo*
16. "first nation"
17. "native american"
18. 6 or 7 or 8 or 9 or 10 or 11 or 12 or 13 or 14 or 15 or 16 or 17
19. 5 AND 18
20. 19 AND transplant*

**ProQuest**

1. haemodialysis OR hemodialysis
2. dialysis OR predialysis
3. kidney
4. renal
5. neph*
6. 1 OR 2 OR 3 OR 4 OR 5
7. indigen*
8. minority
9. ethnic
10. maori
11. "pacific islander*"
12. aborigin*
13. torres
14. hawaii*
15. inuit
16. eskimo*
17. "first nation"
18. "native american"
19. 7 OR 8 OT 9 OR 10 OR 11 OR 12 OR 13 OR 14 OR 15 OR 16 OR 17 OR 18
20. 6 AND 19
21. 20 AND transplant*

**PubMed**

((oceanic ancestry group[MeSH Terms] OR american native continental ancestry group[MeSH Terms] OR ethnic group[MeSH Terms] OR minority group[MeSH Terms] OR minority health[MeSH Terms] OR minority[Other Term] OR ethnic*[Other Term] OR ethnic*[Text Word] OR maori[Text Word] OR first nation*[Text Word] OR native American[Text Word] OR swami[Text Word] OR metis[Text Word] OR inuit*[Text Word] OR pacific islander*[Text Word] OR cook islander*[Text Word] OR aborigin*[Text Word] OR indigenous[Text Word] OR torres strait islander* [Text Word] OR eskimo* [Text Word]) AND (haemodialysis[Text Word] OR hemodialysis[Text Word] OR dialysis[Text Word] OR predialysis[Text Word] OR kidney[Text Word] OR renal[Text Word] OR nephr*[Text Word])) AND transplant*[Text Word])((oceanic ancestry group[MeSH Terms] OR american native continental ancestry group[MeSH Terms] OR ethnic group[MeSH Terms] OR minority group[MeSH Terms] OR minority health[MeSH Terms] OR minority[Other Term] OR ethnic*[Other Term] OR ethnic*[Text Word] OR maori[Text Word] OR first nation*[Text Word] OR native American[Text Word] OR swami[Text Word] OR metis[Text Word] OR inuit*[Text Word] OR pacific islander*[Text Word] OR cook islander*[Text Word] OR aborigin*[Text Word] OR indigenous[Text Word] OR torres strait islander* [Text Word] OR eskimo* [Text Word]) AND (haemodialysis[Text Word] OR hemodialysis[Text Word] OR dialysis[Text Word] OR predialysis[Text Word] OR kidney[Text Word] OR renal[Text Word] OR nephr*[Text Word])) AND transplant*[Text Word])

Filter: MEDLINE 1687

**CINAHL**

( ( haemodialysis OR hemodialysis OR dialysis OR predialysis OR kidney OR renal OR neph* ) AND ( indigen* OR minority OR ethnic OR maori OR "pacific islander*" OR aborigin* OR torres OR hawaii* OR inuit OR eskimo* OR "first nation" OR "native american" ) AND transplant* )

**Embase**

((TI((haemodialysis OR hemodialysis OR dialysis OR predialysis OR kidney OR renal OR neph* ) AND ( indigen* OR minority OR ethnic OR maori OR "pacific islander*" OR “cook islander*” OR aborigin* OR torres OR hawaii* OR inuit OR eskimo* OR "first nation" OR "native american" OR metis OR swami ) AND transplant*)) OR (AB(( haemodialysis OR hemodialysis OR dialysis OR predialysis OR kidney OR renal OR neph* ) AND (indigen* OR minority OR ethnic OR maori OR "pacific islander*" OR “cook islander*” OR aborigin* OR torres OR hawaii* OR inuit OR eskimo* OR "first nation" OR "native american" OR metis OR swami) AND transplant*)) OR (EMB(( haemodialysis OR hemodialysis OR dialysis OR predialysis OR kidney OR renal OR neph* ) AND (indigen* OR minority OR ethnic OR maori OR "pacific islander*" OR “cook islander*” OR aborigin* OR torres OR hawaii* OR inuit OR eskimo* OR "first nation" OR "native american" OR metis OR swami ) AND "kidney transplantation")) OR SU((haemodialysis OR hemodialysis OR dialysis OR predialysis OR kidney OR renal OR neph* ) AND ( indigen* OR minority OR ethnic OR maori OR "pacific islander*" OR “cook islander*” OR aborigin* OR torres OR hawaii* OR inuit OR eskimo* OR "first nation" OR "native american" OR metis OR swami )

AND transplant*))

**Embase (not Medline)**

((TI((haemodialysis OR hemodialysis OR dialysis OR predialysis OR kidney OR renal OR neph* )

AND ( indigen* OR minority OR ethnic OR maori OR "pacific islander*" OR “cook islander*” OR aborigin* OR torres OR hawaii* OR inuit OR eskimo* OR "first nation" OR "native american" OR metis OR swami ) AND transplant*))OR (AB(( haemodialysis OR hemodialysis OR dialysis OR predialysis OR kidney OR renal OR neph* ) AND (indigen* OR minority OR ethnic OR maori OR "pacific islander*" OR “cook islander*” OR aborigin* OR torres OR hawaii* OR inuit OR eskimo* OR "first nation" OR "native american" OR metis OR swami) AND transplant*)) OR (EMB(( haemodialysis OR hemodialysis OR dialysis OR predialysis OR kidney OR renal OR neph* ) AND (indigen* OR minority OR ethnic OR maori OR "pacific islander*" OR “cook islander*” OR aborigin* OR torres OR hawaii* OR inuit OR eskimo* OR "first nation" OR "native american" OR metis OR swami ) AND "kidney transplantation")) OR SU((haemodialysis OR hemodialysis OR dialysis OR predialysis OR kidney OR renal OR neph* ) AND ( indigen* OR minority OR ethnic OR maori OR "pacific islander*" OR “cook islander*” OR aborigin* OR torres OR hawaii* OR inuit OR eskimo* OR "first nation" OR "native american" OR metis OR swami ) AND transplant*))
